# Frequency, determinants and impact of feed intolerance amongst the critically ill

**DOI:** 10.1186/cc10768

**Published:** 2012-03-20

**Authors:** U Gungabissoon, K Hacquoil, C Bains, G Dukes, M Irizarry, D Heyland

**Affiliations:** 1 GlaxoSmithKline, Stockley Park, UK; 2 GlaxoSmithKline, Research Triangle Park, NC, USA; 3 CERU, Queen's University, Kingston, Canada

## Introduction

Provision of early and adequate enteral nutrition (EN) to critically ill patients is associated with improved clinical outcomes; however, 50 to 60% of prescribed EN is received. We aimed to characterise the incidence and determinants of intolerance and assess its influence on nutritional and clinical outcomes using the 2009 Critical Care Nutrition Survey (CCNS).

## Methods

The CCNS survey is a prospective observational cohort study of nutrition practices from over 150 ICUs around the world. Included patients were those that remained in ICU for ≥72 hours and were mechanically ventilated ≤48 hours of admission to ICU. We collected pertinent baseline and outcome data that included nutritional adequacy, ventilator-free days, 60-day mortality and ICU stay. Intolerance was defined as interruption of EN due to gastrointestinal (GI) reasons (high gastric residuals, increased abdominal girth/abdominal distension, vomiting/emesis, diarrhoea or subjective discomfort). In the analysis of intolerance we included each potential effect into a logistic regression analysis to determine its significance.

## Results

Data from 1,888 ICU patients receiving EN were analysed. The incidence of intolerance was 30.5%, and occurred after a median 3 days from EN initiation. Factors associated with intolerance were: diagnosis category (*P *= 0.0009) (GI, cardiovascular and sepsis categories with the highest risk), pre-emptive motility agent use (*P *= 0.0125), non-GI interruptions to feed (*P *= 0.0086) and global region (*P *= 0.0006). Intolerance was associated with poor nutritional adequacy, increased mortality, longer ventilator dependence and increased length of ICU stay (*P *< 0.05) (Table 1). Poorer clinical outcomes were seen with increasing number of days of intolerance.

**Table 1:** Nutritional adequacy and clinical endpoints in tolerant and intolerant EN patients

	% Calorific adequacy	% Protein adequacy	Ventilator-free days	60-day mortality	ICU stay (days)	Time to discharge alive
Tolerant	64.3	63.7	11.2	26.2	11.3	25.2
Intolerant	55.5	55.6	2.5	30.8	14.4	31.1

## Conclusion

Intolerance is common amongst the EN ICU population and is associated with poor nutritional and clinical outcomes.

